# The propensity of fomite spread of SARS-CoV-2 virus through produce supply chain

**DOI:** 10.1186/s42269-022-00935-5

**Published:** 2022-09-16

**Authors:** Akinbode A. Adedeji, Paul Priyesh Vijayakumar

**Affiliations:** 1 Department of Biosystems and Agricultural Engineering, University of Kentucky, Lexington, KY USA; 2 Department of Animal and Food Sciences, University of Kentucky, Lexington, KY USA

**Keywords:** Fomite transmission, SARS-CoV-2, Produce, Supply chain, Coronavirus

## Abstract

**Background:**

The global community has battled the spread of SAR-CoV-2 for almost 2 years, and the projection is that the virus may be recurrent like the seasonal flu. The SARS-CoV-2 pandemic disrupted activities within the food supply chain that cost billions of dollars globally. This has heightened concerns about fomite spread of the virus through surfaces. There is an urgent need to understand the risk portends by this virus along the produce supply chain with conditions (low temperature and high relative humidity) conducive to extended survival of the virus.

**Main body:**

Pre-dating SARS-CoV-2 are other types of coronaviruses that had lower infection and mortality rates. There are some similarities between the former and the new coronavirus, especially with regards to transmission modes and their survivability on surfaces. There is evidence of other coronaviruses’ survival on surfaces for weeks. Currently, there are limited evidence-based studies to enlighten us on how the virus is transmitted within the produce supply chain. A few studies claim that the virus could spread through the cold supply chains. However, these are not sufficient to make a conclusive inference about the deadly SARS-CoV-2.

**Conclusions:**

This paper provides a succinct review of the literature on current understanding of the transmission, survivability, and risk SARS-CoV-2 portend to humans within the produce supply chain and calls for more evidence-based research to allay or alert us of the potential risk of fomite transmission of SARS-CoV-2. The paper also highlights examples of conventional and novel non-thermal inactivation and sanitation methods applicable to this type of virus.

## Background

A direct quote from CDC’s Web site dated April 2021 reads, “It is possible for people to be infected (with SARS-CoV-2) through contact with contaminated surfaces or objects (fomites), but the risk is generally considered to be low.” How low the risk is, we do not know yet because there are no enough evidence-based empirical studies to support or counter this claim. Here is what we know, SAR-CoV-2, the virus that causes COVD-19 continues to pose a serious threat and has caused significant disruption to human activities, including food production since it became a pandemic in 2020. As of June 2022, 192 countries/regions have reported at least one case of the virus infection, and over 535 million cases have been reported globally (that is 294 million more since October 2021) with 85.5 million of those in the USA alone. Over 6.3 million people have died (more than a million in the USA alone as of June 2022) from the virus infection worldwide, with 42,039 deaths alone in the last 28 days (as of June 13, 2022). The mortality rate was about 6% worldwide in June 2020 when testing was not widely available but has dropped to about 2.2% as of July 2021 (Jones et al. 2020; JohnHopkins 2022; WHO 2020). The initial decrease in global infection rate and death can be attributed to several factors such as total lockdown during peak periods, strict social distancing measures implemented, travel restrictions, and the release of effective vaccines across the globe. There is concern that the virus may linger for much longer even with the availability of effective vaccines. This possibility has been attributed to the fact that the vaccine is not available to everyone yet and there is a concern about vaccine hesitancy—some people are not planning to take the vaccine and have not taken it even when it becomes available to their category, and the fact that virus keeps mutating. At some point, about 97% of people who are hospitalized currently in the USA were not vaccinated, the number later decreased to 70% (Olson et al. 2021). Also, many poor countries do not have the resources to procure, distribute and vaccinate all their citizens at the same pace as we have seen in developed countries. The virus continues to mutate and more infectious variants keep coming out. The last two variants are Omicron (B.1.1.529) and Delta (B.1.617.2) which caused a far greater wave of infection and death across the globe, and over 99% of new cases today in the USA are attributed to the former variant (CDC 2021a; Olson et al. 2021). Omicron has been determined to be 50–100% more infectious than Delta, and Delta was found to be about 167% more transmissible than the Alpha (B.1.1.7) variant (B.1.1.7) (Allen et al. 2022; Earnest et al. 2022). More variants of Omicron have since developed that are more contagious but less virulent (BA.1, BA.1.1, BA.2, BA.3, BA.4, and BA.5) (CDC 2022). A parallel has been drawn to why Omicron is more contagious to its longer survivability on surfaces. In a study conducted at the University of Hong Kong, Omicron was found to survive for up to 7 days on stainless steel, polypropylene sheet, and glass at 21–22 °C incubation temperatures against 2 days for the earlier variants (Hong et al. 2022). As of the time of writing the first draft of this paper, October 2021, the USA had experienced four waves of the viruses’ spread and a fifth wave is being predicted if vaccine hesitancy persists. As of today, June 13, 2022, a sixth wave of the infection is being reported across different states in the USA.

One major concern going forward is the fear that the destruction of natural habitats of many exotic animals due to human activities will continue to increase the danger of humans contracting zoonotic and enteric diseases that could constitute a public health catastrophe and concern like SARS-CoV-2 (Lappan et al. 2020; Titanji et al. 2022). It appears the virus is here to stay even beyond when we reach herd immunity, and we know that it is mostly spread by aerosolized droplets (van Doremalen et al. 2020) and in some cases, through surface contact (CDC 2021b; Dietz et al. 2020; Wilson et al. 2021). How much of it is spread through the latter versus the former route and through the food supply chain like the produce (cold) supply chain, so that we can take all steps to reduce the risk, is a question that is begging for an answer.

The spread of SARS-CoV-2 disrupted the global supply chain from production, manufacturing to distribution of products. Food supply, an essential trade, was severely affected at the peaks of the disease’s spread in 2020, especially during the first wave in April–May when the world was yet to understand how to control the virus. The estimated loss to the food industry, particularly the supply chain system from the SARS-CoV-2 spread, is in the hundreds of billions of dollars globally (Aday and Aday 2020). During the first surge of the virus’ spread, the meat processing industry in the USA was so badly affected that many plants were closed down and this caused an increase in the price of meat (McCarthy et al. 2020). The shutdown was predicated on a high rate of infection reported in some meat processing plants. SARS-CoV-2, though a type of coronavirus, its spread, virulence, survivability on surfaces, and transmission appear to be different from the other six coronaviruses before it (McCarthy et al. 2020; van Doremalen et al. 2020). We know that the virus is enteric, and is commonly transmitted through droplets from an infected person. It can also be transmitted from surfaces to humans and vice versa. It can survive on these surfaces (human, paper, plastics, metal, paints, etc.) for a varied period (van Doremalen et al. 2020). However, we do not know enough about how the actual spread occurs within the food supply chain. We do not know how long the virus stays on different surfaces along the food value chain, we cannot tell definitively if humans can contract it from touching contaminated food surfaces in the grocery store, and what type of cleaning regime can effectively sanitize food contact surfaces. The general consensus is that SARS-CoV-2 does not portend any food safety risk through contact with food surfaces, and it is not tagged as a food-borne virus currently (Anelich et al. 2020). This conclusion is mostly based on inference from other coronaviruses. If what we know about the survivability of other coronaviruses is considered, there is a need to be concerned about SARS-CoV-2’s spread through food surfaces. Hence, it is crucial to conduct more evidence-based research that will provide understanding to every stakeholder within the food production and processing sectors, which are among the essential sector, so that measures that will safeguard the spread of this virus are proactively taken. This paper provides a general overview of the origin, transmission, survivability-virulence, and inactivation processes that are being considered in ensuring foods like produce is safe and is not vector for the virus to spread. This paper also points to the fact that we need to do more to know how the virus spreads.

## Main text

### SARS-CoV-2, origin and transmission

**Fig. 1:**
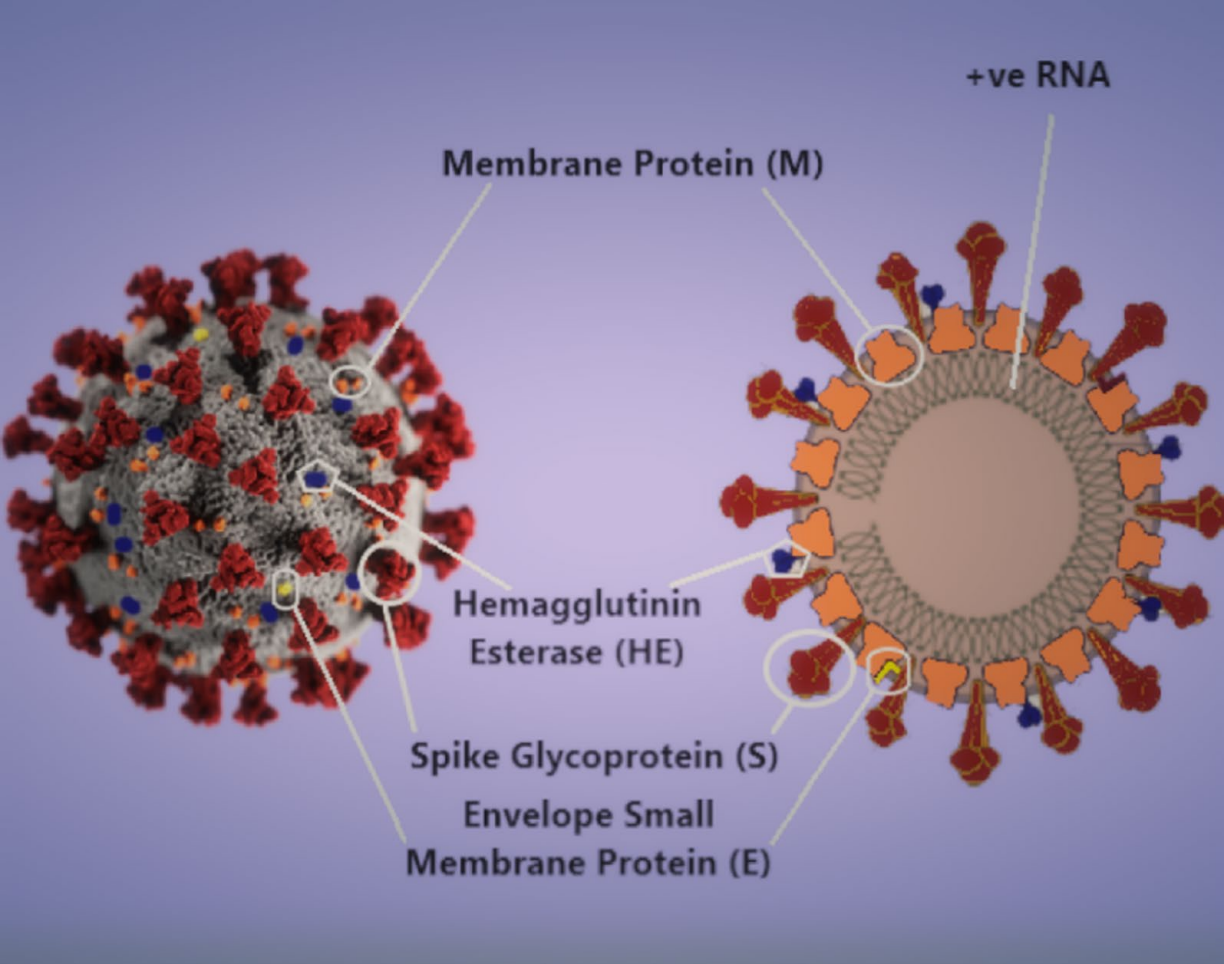
3D model of SARS-CoV-2 virion and a schematic showing its structural proteins and genome. Image parts modified from CDC Public Health Image Library (Eckert and Higgins 2020; Chin et al. 2020; Kakodkar et al. 2020)

Severe acute respiratory syndrome (SARS) is a novel infectious disease that was first reported in November 2002 in China, spreading quickly worldwide, resulting in hundreds of deaths with a 9.6% mortality rate (Kakodkar et al. 2020). Then in 2012, Middle East respiratory syndrome (MERS) was reported in Saudi Arabia, and it has a mortality rate of 34%, with a total of 2494 cases reported worldwide to date (Kakodkar et al. 2020). The current outbreak of coronavirus (SARS-CoV-2/2019-nCoV/novel coronavirus) (Fig. 1) was reported to have originated from Wuhan in the Hubei Province of China, specifically, from the Huanan Seafood Wholesale Market which trades live species of all kinds of wild animals like bats, snakes, pangolins, and badgers (Kakodkar et al. 2020). What is different about SARS-CoV-2 is how it spreads so quickly and its survivability that seems different from the other enveloped coronavirus that made it a pandemic.

Viruses are obligate intracellular parasites because they lack the means for self-reproduction outside a host cell, but unlike parasites, viruses are generally not considered to be true living organisms. They replicate by infecting a host cell. Coronaviruses (CoVs) belong to the subfamily Orthocoronavirinae in the family Coronaviridae, Order Nidovirales. There are four genera in the subfamily Orthocoronavirinae, namely Alpha-coronavirus (*α*-CoV), Beta-coronavirus (*β*-CoV), Gamma-coronavirus (*γ*-CoV), and Delta-coronavirus (*δ*-CoV) (Banerjee et al. 2019; Hakmi et al. 2020). The CoV genome is an enveloped, positive-sense, single-stranded RNA with a size between 26 and 32 kb, the largest genome of known RNA viruses. Both α- and β-CoV genera are known to infect mammals, while *δ*- and *γ*-CoVs infect birds. Coronaviruses are zoonotic enveloped RNA respiratory viruses that rarely transmit between humans in their native form, but could mutate to allow more efficient human-to-human transmission (FDA 2020; Otter et al. 2016). The origin of all coronavirus infections is always traced back to contact between humans and animals that are carriers like bat, pangolin, camel, snake, and other exotic animals. SARS-CoV-2 is linked to some of the animals in the Huanan market. SARS-CoV-2 whole-genome aligned with the genomes of viruses (Bat-CoV and Bat-CoV RaTG13) in *Rhinolophus affinis* species of Yunnan province with 96% similarity (Kakodkar et al. 2020).

In human-to-human transmission, the most frequent routes of spread are droplet transmission, where droplets (> 5 mm diameter, traveling < 1 m) containing viable viruses make contact with the nose, mouth, eyes, or upper respiratory tract, and ‘airborne transmission’, where droplet nuclei (≤ 5 mm diameter, which can travel > 1 m) are inhaled by susceptible individuals (Otter et al. 2016). There is now some evidence that the airborne coronaviruses may reach enough concentration to infect, and the quality of air circulation (heating, ventilation, and air conditioning—HVAC) is critical (EPA 2021). The knowledge of this droplet size–travel distance is the basis for nose mask recommendation as containment and is partially responsible for the six feet distance (Santa-Coloma 2020). Recent data show that six feet recommended by CDC are grossly inadequate to prevent contact with droplets that could travel beyond 2 m (6 feet) (Bahl et al. 2020; Jones et al. 2020). Many studies examined how droplet travels when human sneeze or cough. The studies based on mathematical modeling were found not to be as reliable (Bahl et al. 2020). Where mathematical modeling and experimental validation were combined, results show that aerosolized droplets released during sneezing or coughing could travel as far as 6–9 m (≈ 20–26 feet) (Bourouiba 2021). This lack of consensus on how far the droplets travel and what adequate measures should be taken to decrease the risk of transmission within a closed environment like a food processing plant, mainly by asymptomatic workers, requires more scientific studies.

SARS-CoV-2 and other pathogenic microbes constitute a significant risk even in the most strictly regulated food production environment, especially when someone who has contracted the disease is on the processing floor. Hand sanitization, protective gloves, and goggles may not eliminate the possible spread of an airborne illness among people working in such proximity. The most effective approach is for sick workers to not report for work. In cases of asymptomatic workers, regular testing, effective cleaning, and sanitization\protocols should be in place to ensure foods and the work environment are safe for everyone within the food supply chain. We need to learn what these protocols look like for SARS-CoV-2.

### Survivability of viruses on different surfaces

Survivability, often reported as the time necessary for 90% of a population of microorganisms to lose infectivity, can vary widely among different viruses and depends on environmental conditions, including temperature (Azuma et al. 2020; Memarzadeh 2012), relative humidity (Memarzadeh 2012), radiation (Bosshard et al. 2013) and oxidants (Wigginton and Kohn 2012). There is scientific evidence to show that animal coronavirus remains potent and infectious in water for up to a year depending on the environmental conditions—relative humidity and temperature (Kitajima et al. 2020; Olson et al. 2021). A virus’ structures can also impact its survivability. SARS-CoV-2 is an enveloped virus that is expected to have a short lifespan. However, SARS-CoV-1 is reported to have a life span of 10–15 min at 56 °C, several days at 37 °C and several months at refrigeration temperature, 4 °C and years at subfreezing temperatures like − 60 °C without losing their ability to infect (Chin et al. 2020; van Doremalen et al. 2020). They can survive in saline solution for up to 6 days at room temperature but have a shorter life span (about 3 h) on dry surfaces depending on the type of surface (Andries et al. 1978; Ijaz et al. 2016). Table 1 shows how a combination of relative humidity (RH) and temperature impacts the survival rate of HCoV 229E, a type of human coronavirus. A study reported that SARS-CoV-2 survives at low temperatures on inert surfaces than at high temperatures (Morris et al. 2020). They reported 24 h as the median half-life of SARS-CoV-2 at 10 °C and 40% RH but was one and half hours at 27 °C and 65% RH. Depending on the particular type of coronavirus, differences were observed in how long they survived on different surfaces (Sizun et al. 2000). van Doremalen et al. (2020) reported that SARS-CoV-2 is more stable on plastic and stainless steel than on copper and cardboard. They found a viable virus on the latter’s surface after 72 h, whereas beyond 4 h on copper, nothing was detected. These observations suggest that surface characteristics aside from the environmental conditions play a significant role in how coronavirus binds and survives on surfaces.

**Table 1:** Survival rate of HCoV 229E: a type of human coronavirus. *Source* (Geller et al. 2012)

Temperature	20 °C				6 °C	
Relative humidity (%)	15 min (%)	24 h (%)	72 h (%)	6 days	15 min (%)	24 h (%)
30	87	65	> 50	n.d	91	65
50	91	75	> 50	20%	96	80
80	55	3	0	n.d	105	80

*n.d.* not done

Low-temperature storage, high humidity and surface characteristics of fruits and vegetables, especially fresh-cut fruits and rough surface vegetables, may predispose them to sustain and be conducive to microbial attachment and growth (FDA 2015). SARS-CoV-2 is reported to survive on smooth surfaces (stainless steels for food equipment, apples, tomatoes, etc.) as well as rough surfaces such as those of fruits and vegetables (e.g., pineapple, cantaloupe, leafy vegetables like cabbage, lettuce, etc.) (Chin et al. 2020). Rough surface configuration as found in produce like lettuce and cantaloupe is reported to increase microorganisms' survivability (Mullis et al. 2012). This makes this type of produce often consumed raw at risk of being a vector for lethal microbes like SARS-CoV-2. Rough surfaces typically have higher surface areas than smooth surfaces of the same dimension. The grooves and roughness are reported to help accommodate the growth of food-borne microorganisms (Mullis et al. 2012; Yu et al. 2016). Thus, contaminated ready-to-consume produce may be potential vehicles for enteric transmission of SARS-CoV-2 to humans. There is scientific evidence that this virus is capable of tuning itself to effectively adhere to any surface (Pandey 2020), which is more of a reason for a scientific understanding of how this virus cleaves and survives on surfaces.

The transmission mode and the survivability of SARS-CoV-2 on surfaces make them a risk factor in food processing plants where workers repeatedly touch produce and packaging materials. Currently, there is minimally verifiable scientific evidence based on empirically collected data to inform us how SARS-CoV-2 binds and survives on different surfaces for us to make a definitive conclusion that SARS-CoV-2 cannot be transmitted through other means other than aerosolized droplets, that fomite transmission (especially food and food processing system surfaces) can be ignored. Based on our knowledge of transmission and survivability of viruses, contamination of food and food systems surfaces like the packaging materials by the virus as a consequence of human handling can occur at any stage of food production or processing (Boxman 2013). Liu et al. (2020) reported that SARS-CoV-2 nucleic acids were detected on 50 out of 421 surfaces tested and they successfully matched the genetic composition of these positive tests to throat swab from two asymptomatic stevedores who were solely responsible for loading and unloading of contaminated cod fish packaging. Based on this evidence, they concluded that there is a likelihood that these workers contracted the virus from the frozen packages’ surfaces. They also reported that no live virus was isolated due to low nucleic acid concentration in the tested samples. Between July 2020 and January 2021, several incidents (Wilson et al. 2021) of SARS-CoV-2 infections were reported at various ports in China, including Qingdao port, and investigation showed frozen food, packaging, and food processing surfaces (cutting board) tested positive for the virus (Han and Liu 2021). They referred to claims that SARS-CoV-2 can survive up to 21 days under cold temperatures (< 0 °C) (Chi et al. 2021; Aboubakr et al. 2021; van Doremalen et al. 2020) as a reason for the detection of the virus after a long storage period.

Most of the other studies on fomite transmission are based on modeling and projections. Using the quantitative microbial risk assessment modeling (QMRA) approach, Pitol and Julian (2021)) reported that the risk of surface transmission is between 0.2 and 5% (< 10^−6^) using. Sobolik et al. (2021) also reported that the risk of fomite transmission of the virus in the cold chain for workers in contact with contaminated packaging was 2·8 × 10^−3^ per 1 h-period (95%CI: 6·9 × 10^−6^, 2·4 × 10^−2^). They reported that the risk was reduced by about 10^−6^ (0.3% infection rate) when standard infection control measures (vaccination, handwashing, and masks) were implemented. These projections were based on the QMRA modeling approach and not actual empirical study. Inferring from models without validation and other coronaviruses could be misleading based on evidence from differences in the behavior of other types of coronavirus in this context (Otter et al. 2016). This is the reason why studies must be conducted to fill this knowledge gap (Han and Liu 2021).

### Risk posed by SARS-CoV-2 to agricultural produce supply chain

**Fig. 2:**
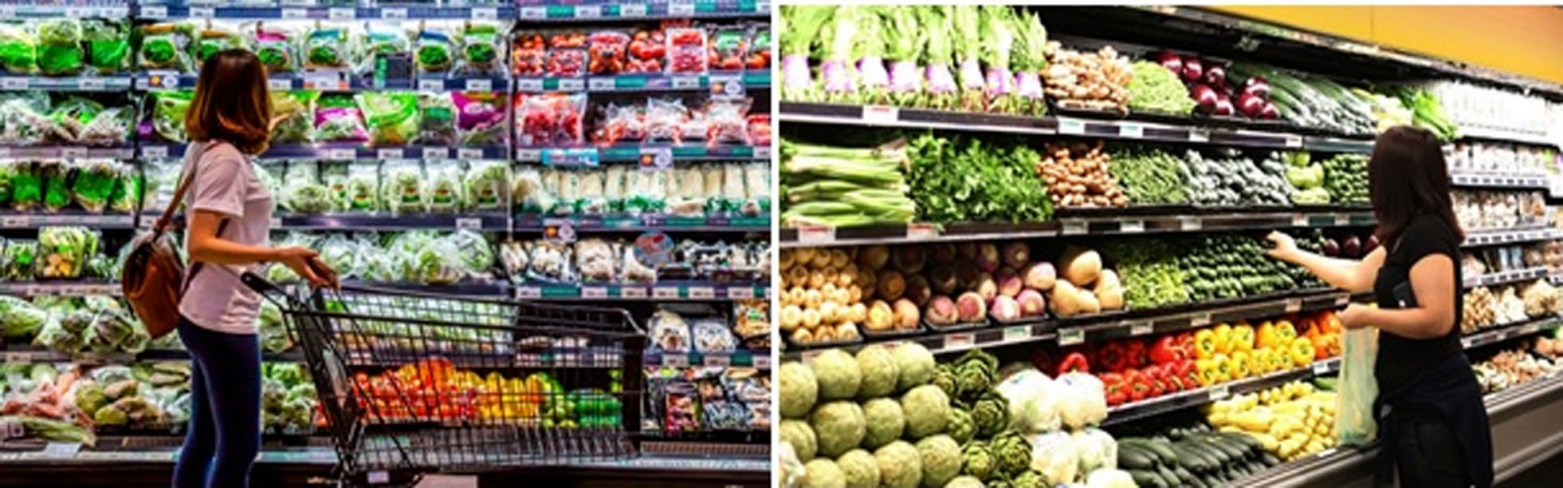
Typical grocery settings for customers. Source of images: Left-(Waldbieser 2019) and Right-(Fung and Haddon 2019)

COVID-19, the disease caused by the SARS-CoV-2 virus, could be a potential food-borne disease even though CDC and FDA say there is no evidence that the virus can be contracted from food (CDC 2020; FDA 2015). While the acidic nature of the human gastrointestinal tract may not be conducive to SARS-CoV-2 survival, the risk posed by food and food systems surfaces becoming a vector for the virus transmission to humans when touched is high. There is evidence that other coronaviruses constitute a big danger to consumers through transmission from surfaces like food packaging (Geller et al. 2012; Mullis et al. 2012; Sánchez and Bosch 2016). The fact that SARS-CoV-2 can survive for a long time in a humid environment similar to produce and for an extended time on different food contact surfaces in grocery stores (Fig. 2) implies that carefully delivered science be brought to play to address the question of survivability. Ijaz et al. (1985) reported that enveloped viruses like human coronavirus survive for as long as 6 days in wet conditions as you find in many fruits and vegetables. Mullis et al. (2012) examined the stability of bovine coronavirus on lettuce and demonstrated that the virus was stable during the shelf life of romaine lettuce, and the elution washing process did not completely remove the residual virus. This understanding informs the reason why there is a need for a definitive study to clear the air on SARS-CoV-2 survivability on food and food system surfaces.

**Fig. 3:**
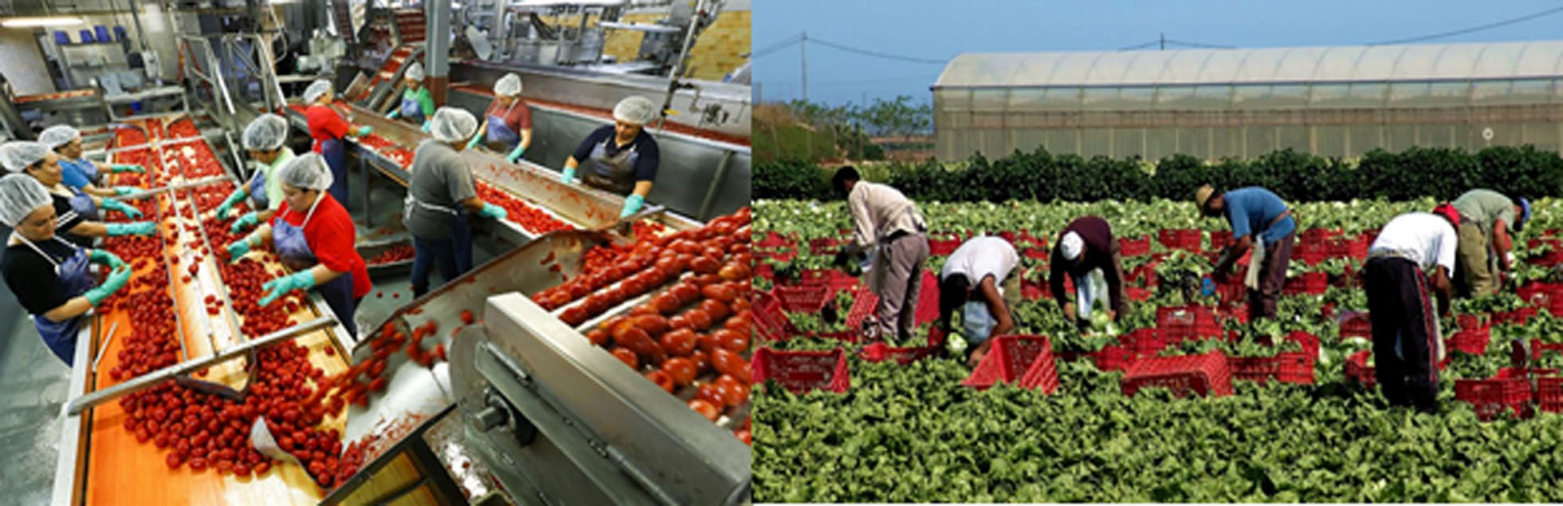
Tomato processing plant (left) (Linkhorn 2016) and lettuce picking farm (right) (Verite 2020)

The known transmission modes for infectious microbes like viruses generally are zoonotic (animal-to-human) and enteric (human-to-human transmission) common where humans work in proximity to one another (Fig. 3). Because of human contact with surfaces during food processing, there is a high possibility of human-to-surface transmission. Even after the cleaning and sanitization regimes, we are not sure of the most efficacious method against SARS-CoV-2 right now. There is still the possibility of transmission of the virus to food through the packaging material. SARS-CoV-2 is said to have a different survival rate depending on the type of source (Mullis et al. 2012). Most of the critical information is inferred. There is no clarity on how long SARS-CoV-2 stays on surfaces—human hands or different food surfaces.

Food processing plants (farms) require that workers wear protective gear such as disposable coverall gowns (made from cloth or plastic), hand gloves, and sometimes, a nose mask, as shown in the images in Fig. 3. This is not a mandatory requirement in some processing plants. With the current situation, direct contact with produce may predispose them to become a vector for SARS-CoV-2. Fedio et al. (2012) reported that produce is often the vehicle for transmission of many food-borne pathogens, particularly sprouts with rough surfaces.

A lot of the processing in the food processing plants involves significant water use in a high humidity environment. Viruses are known to survive in water or high humidity conditions for a much longer time than in dry conditions (Geller et al. 2012). Part of what is not clear is how long does SARS-CoV-2 survive on foods of different surface texture. We do not know how wet conditions and relative humidity affect the survivability of the virus.

### Cleaning and inactivation of pathogenic microorganisms from food and food system surfaces

SARS-CoV-2 is a type of enveloped virus mostly enteric because it is transmitted via aerosol (droplets) and from human to human or by human contact with contaminated surfaces or vice versa. Its counterpart, SARS-CoV-1 is reported to have a shorter life span than non-enveloped viruses but SARS-CoV-2 seems to show survivability similar to non-enveloped viruses lasting for days or months depending on the environmental conditions (Rabenau et al. 2005; Ye et al. 2018). Despite SARS-CoV-1 higher environmental stability compared to the previously characterized human coronavirus (HCoV-229E), it can be easily inactivated thermally and chemically. Non-thermal methods have the merits of preserving most of the nutrient composition of food and leaving no residual chemicals, which are often preferred in microbial inactivation. However, the effectiveness of any method, physical (UV light), or chemical agent or combination of both for inactivating a virus is based on how they can target the proteins or the genome of the virus (Ye et al. 2018) and what impact they have on the quality of the food being treated.

Ultraviolet (UV) energy application in inactivating microbial cells has grown significantly in the last two decades. UV treatment, around the far UV end of the electromagnetic spectrum (200–280 nm), is an effective non-thermal treatment to reduce the microbial load in fruits and vegetables by damaging the microbes’ DNA (Fernández-Suárez et al. 2013). The region is designated as UVC and its radiation leads to direct photolysis of photolabile virus components regardless of their solvent accessibility. A study showed that UVC fluence > 1 J/cm^2^ is effective in inactivating SARS-CoV-2 on N95 masks but it took about 62 min (Card et al. 2020). Pulsed UV light, a more rapid method that delivers a large dose of photoenergy intermittently to a surface within a few seconds, could be explored in the inactivation of SARS-CoV-2 because exposure of produce to UVC light for an extended time could lead to a significant increase in temperature that will cause undesirable changes in produce. The generation of light pulses is carried out by the excitation of inert gases, like xenon in flash lamps, and the collision of gaseous molecules due to electrical pulse application. The light energy is then released in the form of short-duration light bursts in a highly concentrated manner (lasting for a few hundred microseconds, usually 1–100 μs). Pulsed UV light is capable of deactivating DNA by photochemical, photothermal, and photophysical methods. It is classified as a sterilization agent, and FDA determined it to be safe for food treatment (Mandal et al. 2020).

Cold plasma can be characterized as partially ionized gas and is a complex mixture of different components, such as charged particles (electrons and ions) and neutral species (atoms and molecules), in addition to radicals, UV photons, and irradiated heat. They are classified as thermal and non-thermal/cold plasmas. Cold (atmospheric) plasma generates partially ionized high-level bactericidal molecules (> 100 ppm ozone, nitric oxides, peroxides, etc.) with minimal power under room temperature conditions in seconds to minutes, with little or no product heating. The temperature of the electrons it produces is in the range of several thousand Kelvin, but the temperature of the neutral species and ions is close to ambient temperature. This is the form most attractive for microbial inactivation on produce often eaten raw. Cold plasma inactivates microbes by causing cell lysis with the aid of the reactive species that it produces, causing the formation of volatile compounds via chemical reactions and openings and lesions in the cell membrane causing electrostatic disruption, DNA damage, and lipid and protein oxidation (Hertwig et al. 2018). Xia et al. (2020) reported a 2.3 log CFU/cm^2^ reduction in the viral *(*bacteriophage MS2*)* population. Kilonzo-Nthenge et al. (2018) showed a 5.5log_10_ CFU/cm^2^ of *Salmonella* and *E. coli* on apples. Cold plasma efficacy on SARS-CoV-2 on food is yet to be tested and reported.

The pH of water used for processing produce ranges from 6.5 to 7.5 (McGlynn 2016). Hypochlorite (HOCl) solution at a concentration as high as 200 ppm is often used in produce sanitation to remove all microbes of public health significance. The US Federal regulations (21 CFR Part 173) on secondary direct food additives permitted in food for human consumption specify the condition for the use of HOCl. Its concentration must not exceed 2000 ppm, and the produce must be thoroughly washed after treatment. In a study by Ukuku et al. (2018), they found that 200 ppm (mg/L) concentration of HOCl completely removed *Salmonella*, aerobic mesophilic bacteria, yeast and mold, and *Pseudomonas* cocktails up to 4.5 log_10_ CFU/cm^2^ from melon and cantaloupe after storage at 22 °C for up to 5 h. It is not enough to infer from studies that assessed other microbes like Salmonella or other coronaviruses to develop an efficient cleaning regime for SARS-CoV-2 because the virus activities so far have proven that control steps are supposed to be based on direct sound science to curb its spread.

Other treatment regimens that have been found to be effective against other types of coronaviruses include ozone treatment and chlorine (Kitajima et al. 2020). The extent to which these treatments work on SARS-CoV-2 is not known, the more reason why adequate studies that determine effective regimes that can keep everyone safe within the produce value chain needs to be carried out.

## Conclusions

This paper captures the state of our understanding of the possible fomite spread of SARS-CoV-2, especially through the agricultural produce supply chain. It provides a scientific report and review of studies on the virulence, survivability, transmission, and risk portend by this virus to the agricultural produce supply chain, mostly drawing inferences from similar coronaviruses and a few studies available so far, mostly based on models. While there is no conclusive evidence yet that this deadly virus can be transmitted from food surfaces to human, or vice versa, the enteric nature (confirmed surface to human transmission) of this virus and what we know about other coronaviruses, and the fact that there are new strains of SAR-CoV-2 that are more contagious calls for research that will ascertain that there is no risk. Despite many being vaccinated, it is evident that the antibodies’ effect wanes after a few months. The duration of the booster shot newly approved is not known. It is obvious that coronavirus may be a major public health concern for many more years. Beyond the vaccine treatment, the food industry needs to devise means to ensure its spread is curbed within the food supply chain. More important is the preparedness needed by the food industry to curtail the possible future spread of any type of enteric and highly contagious microbe like SARS-CoV-2 within the food supply chain (Ijaz et al. 2020). There is a need for stakeholders within the food industry in different countries and regions to continue to engage regulatory agencies and government officials who influence policy to ensure that effective control and proactive measures are in place to curb mutation and future occurrence of organisms of public health concern of this nature.

## Data Availability

The COVID-19 global dataset presented in this manuscript is available at the Center for Systems Science and Engineering (CSSE) at Johns Hopkins University (JHU): https://coronavirus.jhu.edu/map.html.

## Abbreviations

SARS-CoV-2: Severe acute respiratory syndrome coronavirus-2
HCoV 229E: Human coronavirus 229E
HOCl: Hypochlorite
CFU: Coliform-forming units
UVC: Ultraviolet light C
CDC: Center Disease Control and Prevention
